# Frizzled-10 and cancer progression: Is it a new prognostic marker?

**DOI:** 10.18632/oncotarget.23159

**Published:** 2017-12-12

**Authors:** Maria Principia Scavo, Livia Fucci, Lucia Caldarola, Anita Mangia, Amalia Azzariti, Giovanni Simone, Giampietro Gasparini, Silke Krol

**Affiliations:** ^1^ Translational Nanotechnology Laboratory, IRCCS Istituto Tumori “Giovanni Paolo II”, 70124 Bari, Italy; ^2^ Pathology Laboratory, IRCCS Istituto Tumori “Giovanni Paolo II”, 70124 Bari, Italy; ^3^ Functional Biomorphology Laboratory, IRCCS Istituto Tumori “Giovanni Paolo II”, 70124 Bari, Italy; ^4^ Experimental Pharmacology Laboratory, IRCCS Istituto Tumori “Giovanni Paolo II”, 70124 Bari, Italy; ^5^ Direzione Scientifica, IRCCS Istituto Tumori “Giovanni Paolo II”, 70124 Bari, Italy; ^6^ Fondazione IRCCS Istituto Neurologico “Carlo Besta”, 20133 Milan, Italy

**Keywords:** colon cancer, melanoma, gastric cancer, frizzled-10, prognostic marker

## Abstract

Frizzled (FZD) proteins, a family of Wnt receptors, are involved in carcinogenesis in different organs. One interesting FZD protein is FZD-10 highly expressed in embryogenesis but completely absent in the membrane or cytosol of healthy proliferated cells. We studied in detail the expression level and the location of Frizzled-10 protein in different cancerous tissues, such as colon, melanoma and gastric cancer and in function of different staging of the tumor and in metastases. We observed a correlation between cancer evolution and FZD-10 expression, and localization of protein during carcinogenesis. In colon, we have an increase of cytoplasmic FZD-10 expression from hyperplastic mucosa to metastatic tissues, while the amount in the nucleus decreases significantly in T3 and T4 staging tumors as well as in metastases. In melanoma and gastric cancer, we observed the opposite trend of FZD-10 protein in the cytosol but both show a decrease in the T3 and T4 stage of the tumor and in metastases. However, the decrease is less prominent in gastric cancer.

Our findings indicate an important role of FZD-10 in tumor progression especially in the later stages of tumor. The nuclear expression of FZD-10 or its absence can give a new tool for tumor staging to pathologists. For target therapy, at least for colon cancer, the high presence of FZD-10 in the later stages of tumor progression and the absence in healthy tissue present a promising new approach.

## INTRODUCTION

The Wnt signaling pathway is an evolutionarily conserved pathway, which regulates cell processes such as cell migration, cell polarity, neural patterning and organogenesis during embryonic development [1]. Dysregulation of Wnt signaling has adverse consequences on the developing embryo. Moreover, it was identified as an incident factor for some pleiotropic human pathologies, such as colon, skin, breast and glia cancers, skeletal defects and human birth defect like spina bifida [2]. In health, Wnt binds to the N-terminal extracellular domain rich in cysteine of the Frizzled (FZD) protein receptor [3]. The 10 FZD proteins are seven-transmembrane-span protein with topological homology to G-protein coupled receptors. It interacts with co-receptor proteins or other cytoplasmic proteins like low-density-lipoprotein-related protein 5/6 (LRP5/6) and dishevelled phosphoprotein (Dsh/Dvl) [4]. After the binding of Wnt to the receptor complex, the signal is transduced to cytoplasmic phosphoprotein Dsh/Dvl. Previous studies showed that FZD can also interact directly with Dsh [5]. However, the role of some FZD proteins during the carcinogenesis is still unclear.

We will focus our attention on FZD-10. FZD-10 protein was discovered in cancer tissue in 1999. The nucleotide sequence analysis showed that human FZD-10 gene encodes a seven-transmembrane-receptor of 581 amino acids, with the N-terminal cysteine-rich domain and the C-terminal Ser/Thr-Xxx-Val motif [6]. FZD-10 is usually located in plasma membrane and the expression of this protein is almost absent in all types of fully developed healthy organs [7]. These two criteria makes FZD-10 a good target for antibody therapy for colon cancer in particular and in cancer in general and a new tumor marker in case of uncertain diagnosis [7–9].

In the present study, we analyzed expression patterns and localization of FZD-10, in adenocarcinomas of colon, skin, and stomach. We examined the relationship between expression and localization of FZD-10 in carcinogenesis and poor diagnosis.

## RESULTS

### Colon

We analyzed the FDZ-10 signal and distribution according to the pathologic disease staging and compared the FDZ-10 distribution with hyperplastic colon tissue. For 3 patients for which we had tumor tissue (1 patient with T1 cancer classification and 2 with T2 cancer classification) as well as polyps with both low-grade dysplasia and high-grade dysplasia we compared the FDZ-10 level and distribution for those three tissues to exclude inter-patient variations. The patient data and the staging can be found in Supplementary Table 1 in Supplementary Material.

**Figure 1:**
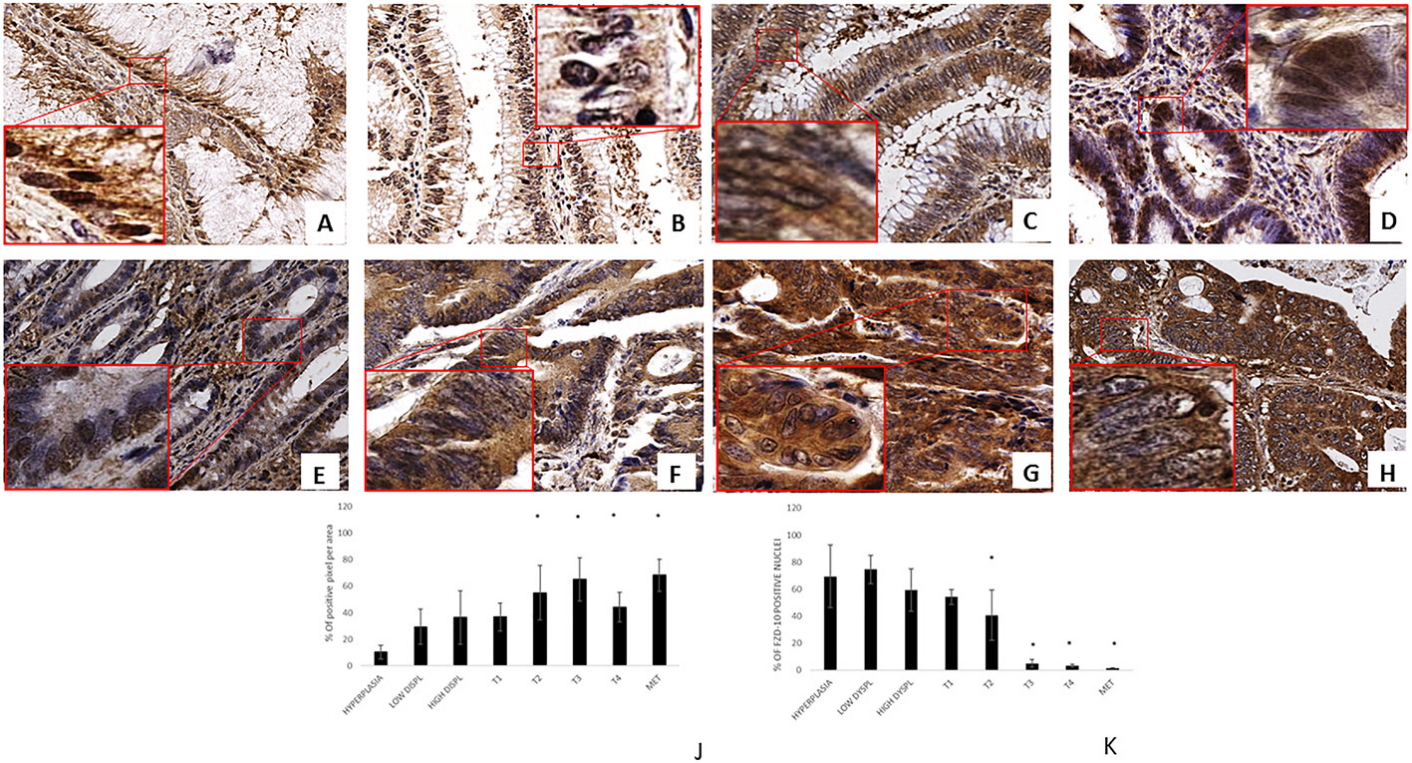
FZD-10 expression in hyperplastic, dysplastic and carcinomatous colon tissues from patients with sporadic cancer Representative images of the immuno-histochemical expression of FZD-10 in hyperplastic mucosa (**A**), adenomas with low-grade dysplasia (**B**) and high-grade dysplasia (**C**), in carcinomatous tissue, T1 (**D**), T2 (**E**), T3 (**F**), T4 (**G**) and metastases (**H**) (×40 magnification). Insets show magnified nuclei for selected areas (squares). The diagram in (**J, K**) shows the percentage of FZD-10 positive pixels per area in J reported as the mean ± SD obtained for the colon tissue of 19 patients and in K the % of positive nuclei in 10 different area of sample: (J) protein expression in cytoplasm and membranes, (K) Nucleic protein expression. Significance was determined by ANOVA test [*p* < 0.001(^*^) and *p* = 0.05] by the Holm–Sidak method.

Figure 1A–1H show representative images with a distribution of FZD-10 in tissue of patients diagnosed with different stage of sporadic colon cancer. In the insets, the magnification shows the nuclei of selected areas to highlight the distribution of FZD-10. It can be clearly seen that in hyperplasia the level of FZD-10 is lower (light brown) in membrane and cytosol as compared to other tested tissues and a strong brown signal of FZD-10 is co-localized with the blue staining for the nucleus of the cells. The same is true for the localization of the FZD-10 signal in low- and high-grade dysplastic tissue with an increase of FZD-10 observed in the cytosol and membrane. The trend is more prominent after image analysis with focus on the location of the FZD-10 (Figure 1J, 1K). Comparing “normal” mucosa (hyperplasia) with adenomatous tissues with low- and high-grade dysplasia, carcinomatous tissues, in different staging and colon hepatic metastasis, we observed a significant increase (expressed in % of number of positive pixel) in FZD-10 expression in both cytoplasm and membrane regions from hyperplasia (10.45% ± 5.12) to T2, T3, T4 and metastatic tissue (55.06% + 20.47, 65.18% + 16.29, 44.25% + 11.21, 68.24%+ 12.23, respectively), (Figure 1J) while no strong differences in its expression levels among the different stages of the colon tumors were evident. The FZD-10 expression in hyperplastic sample as compared to T1 tissue was also not statistically significant different. More interestingly, we observed a significant reduction of this protein in the nucleus comparing hyperplastic tissue with pathological tissue (T2, T3, T4 and metastatic tissue) with a decrease of FZD-10 positive nuclei from about 78.73% to 40.67% ± 18.65, 5.084% ± 2.96, 3.389% ± 1.24, 1.694% ± 0.053, respectively (Figure 1K). A summary of the analysed tissues and the FZD-10 distribution can be found Supplementary Table 2.

**Figure 2:**
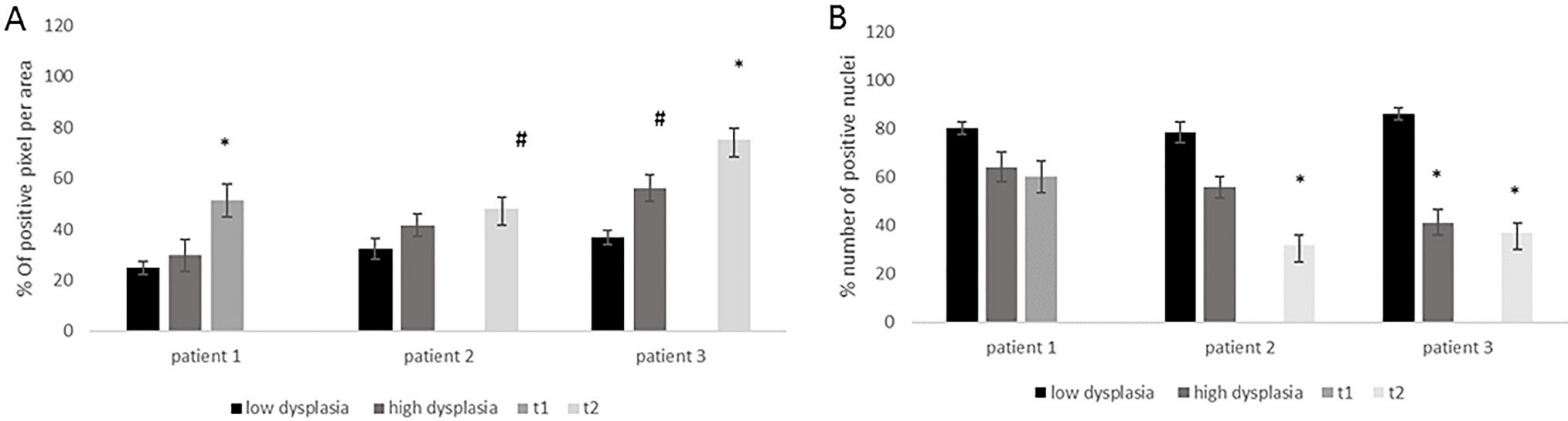
The diagrams show the protein expression in the cytoplasm (**A**) and the nuclei (**B**) in % of FZD-10 positive pixels per area for different grade of disease from the same patient (*n* = 3) and % of positive nuclei in 10 different area of sample. The statistical significance was determined by ANOVA test [*p* < 0.001(^***^) and *p* = 0.05(^*^)].

The possibility to analyze from the same patient different stages of malignancy with synchronous adenomatous polyps, and adenocarcinoma allowed excluding individual and environmental variability factors (like different diet, different collateral disease, sex). Therefore, we analyzed for 3 patients the FZD-10 level in dysplastic and malignant tissue. For cytoplasmic and membrane expression levels we detected a continuous increase with severity of malignancy (T1: 51.4 ± 6.58; low dysplasia: 24.9 ± 2.5 in patient 1). The same trend was observed for the other two patients with T2 carcinoma compared with low dysplasia (patient 2: T2:48.2 ± 4.44; low dysplasia: 32.43 ± 4.15 with *p* = 0.05; patient 3: T2: 75.1 ± 6.71; low dysplasia: 36.8 ± 2.64 with *p* < 0.001) (Figure 2A). The trend of decrease in the nuclear expression of FZD-10 with increasing malignancy observed in the average of 28 patients was also found for the single patients (patient 2: T2: 31.6 ± 2.78 to low dysplastic tissues: 78.4 ± 8.4 patient 3: T2:36.8 ± 4.9 to low dysplastic tissues: 86 ± 7.2; Figure 2B). Interestingly the expression of FZD-10 in the nuclei remains almost at same level in the patient with stage T1 cancer (60.1 ± 3.45 Vs 80 ± 5.89). To summarize we found a significant increase of FZD-10 expression in membrane and cytoplasm from non-dysplastic to dysplastic and to malignant tissue and significant reduction in nuclei expression with a threshold at T2 by analyzing the tissue samples with the Holm–Sidak method.

### Skin

**Figure 3:**
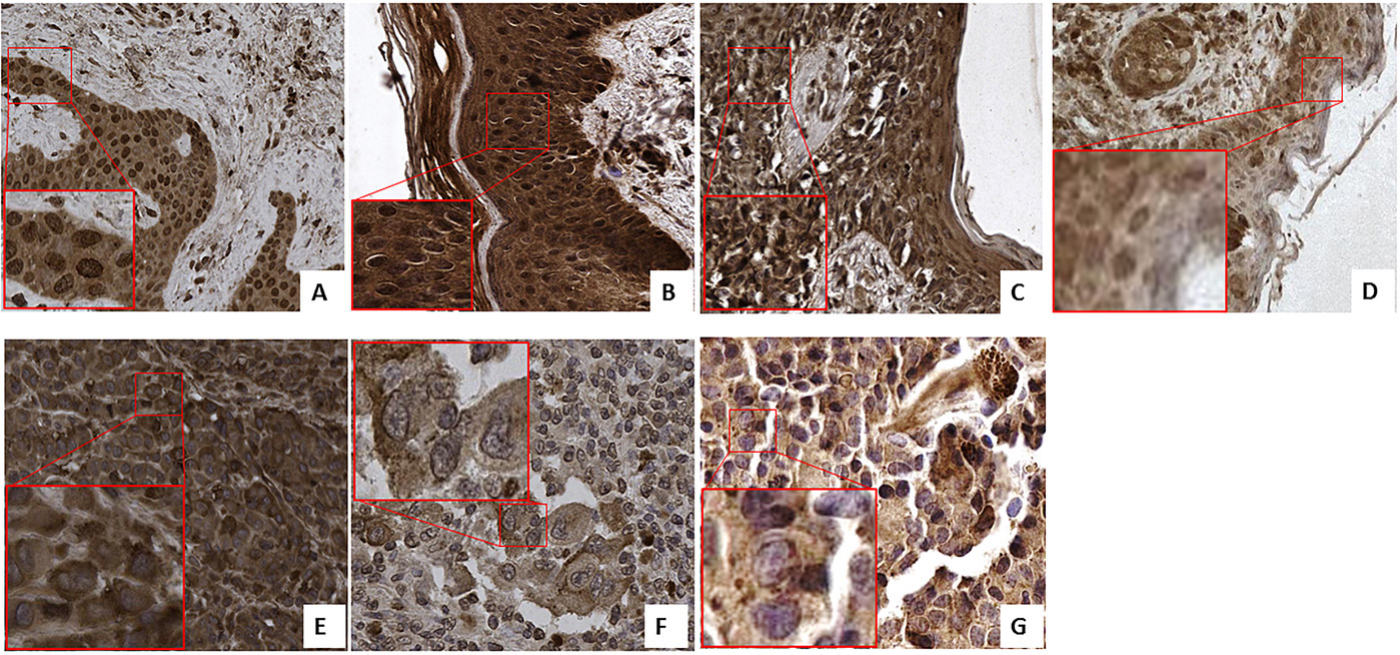
FZD-10 expression in dysplastic skin and melanoma tissues Representative images of the immuno-histochemical expression of FZD-10 in hyperplastic tissue (**A**), *in situ* Tumor (**B**) and in melanomatous tissue, T1 (**C**), T2 (**D**), T3 (**E**), T4 (**F**) and metastases (**G**) (×40 magnification). Insets show magnified nuclei for selected areas (squares).

**Figure 4:**
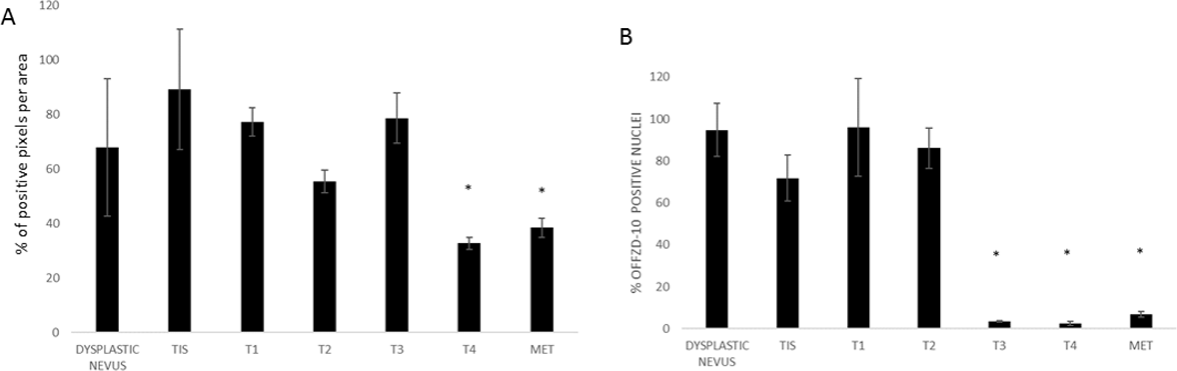
The diagram shows the percentage of FZD-10 positive pixels or positive nuclei per area reported as the mean ± SD obtained for skin tissue samples of 29 patients: (**A**) protein expression in cytoplasm and membranes, (**B**) Nucleic protein expression. The statistical significance was determined by ANOVA test [*p* < 0.001(^***^) and *p* = 0.05(^*^)] and by the Holm–Sidak method.

We selected the tissue of patients from 7 distinctive groups according to the stage and characteristics of the disease as described in Supplementary Table 1 in Supplementary Material: 7 dysplastic nevi, 3 Tis, 6 T1, 3 T2, 3 T3, and 1T4 melanoma and 6 lymph nodes metastases. In contrast to the strong increase in FZD-10 expression on the membrane and cytosol in the colon, we observed a significant decrease (*P* < 0.001) comparing the dysplastic tissue (expressed in % of number of pixel) (67.89% ± 25.23) with the advanced stages of tumor T4 and metastases (32.64% ± 2.23 and 38.42 ± 3.58, respectively) (Figure 3) while the dysplastic tissue and tumor levels up to T3 showed comparable FZD-10 levels. (Figure 4A). However, the trend we observed for the FZD-10 expression in the nucleus was also observed for skin cancer with a strong significant decrease for T3, T4 and in metastatic lymph nodes tissues, as compared to dysplastic tissue (respectively 3.24 ± 0.26 in T3, 2.56 ± 1.1 T4 and 6.89 ± 1.45 in metastasis Vs 94.7 ± 12.56 in dysplastic tissue) (Figure 4B).

### Gastric tissue

**Figure 5:**
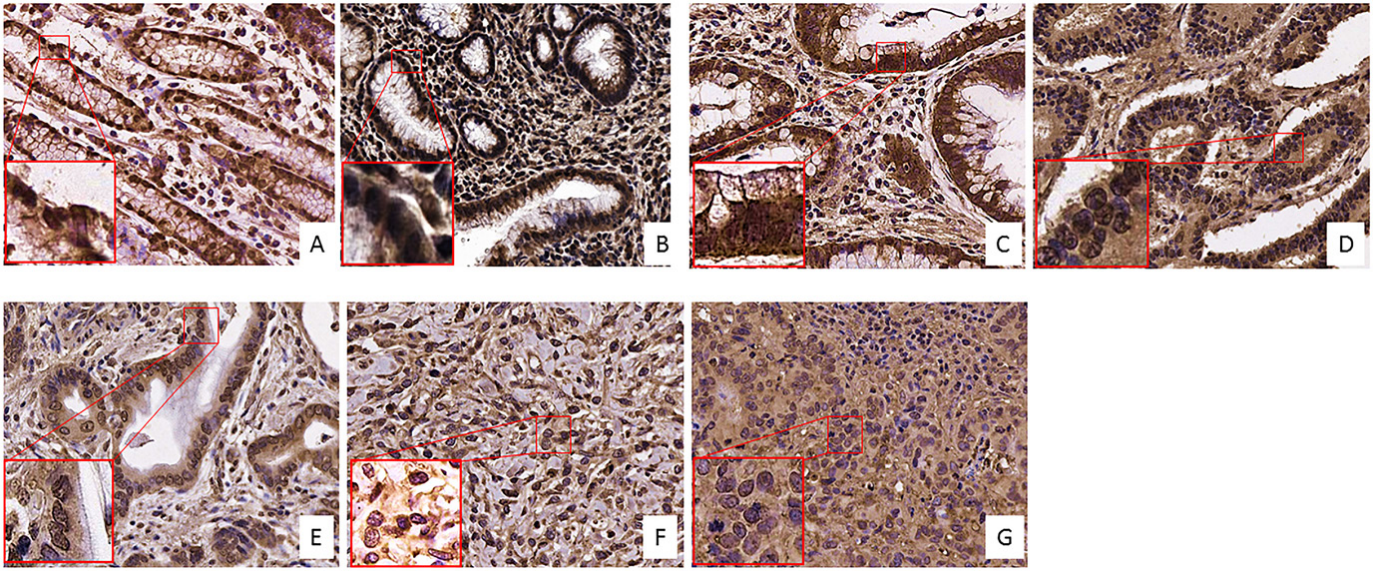
FZD-10 expression in dysplastic and tumor gastric tissues Representative images of the immuno-histochemical expression of FZD-10 in low-grade dysplasia tissue (**A**), high-grade dysplasia (**B**), in carcinomatous tissue, T1 (**C**), T2 (**D**), T3 (**E**), T4 (**F**) and metastases (**G**) (×40 magnification).

**Figure 6:**
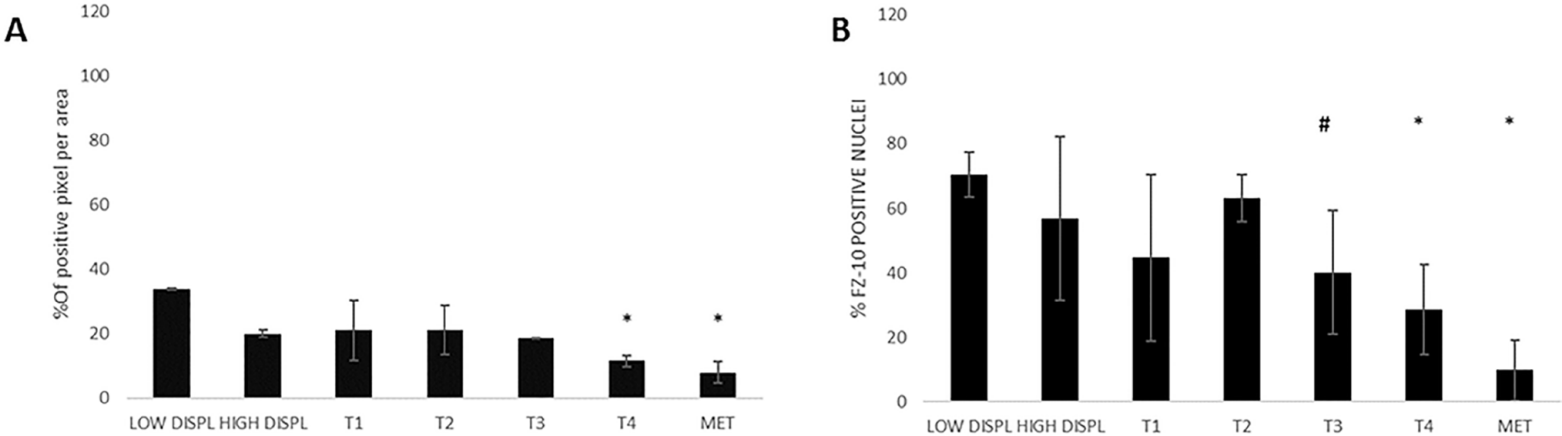
The diagram shows the percentage of FZD-10 positive pixels or positive nuclei per area reported as the mean ± SD obtained for 20 samples of gastric tissue of patients: (**A**) protein expression in cytoplasm and membranes (**B**) Nucleic protein expression. The statistical significance was determined by ANOVA test [*p* < 0.001(^***^) and *p* = 0.05(^*^)] and by the Holm–Sidak method.

For the study on gastric cancer, we selected again tissues from 7 distinctive groups according to the stage and characteristics of disease: 2 low dysplasia, 2 high dysplasia, 5 T1, 6 T2, 2 T3, 3 T4 and 4 metastases. From our results, it has to be stated that the expression level of FZD-10 in gastric tissue samples was lower than in colon and in skin tissues. However, we observed a continuous decrease of FZD-10 expression in the cytosol, with a significant value (*P* < 0.001), from T4 and metastasis to low dysplasia (11.5 ± 1.77 and 7.99 ± 3.33 Vs 33.7 ± 0.25 respectively) with a protein localization in the membrane only in the early stage of disease (T1 and T2) (Figures 5 and 6A). As already determined in other tumors, the nuclear expression of FZD-10 in T3, T4 and metastatic tissue showed a significantly decrease as compared to low dysplasia (40.11 ± 19.22, 28.606 ± 13.89, 9.82 ± 9.25, Vs 70.47 ± 7.07, respectively) (Figure 6B). However, we found also larger variations in the values for the single samples as it can be seen by the relatively big error.

## DISCUSSION

The role of some Frizzled receptors is still not completely understood. One of the most promising receptors in terms target therapy is FZD-10 as it was found to be not expressed in healthy fully proliferated tissue but highly expressed in cancerous tissue. Most studies focus on a general expression profile of FZD-10 in tumor tissue, mainly in colorectal cancer [10]. In the presented study, we investigated a more detailed cellular distribution of FZD-10 in three different types of pathological tissue (colon cancer, melanoma and gastric cancer) and for different pathological stages (low and high dysplasia in colon and gastric tissue, tumor *in situ*, different cancer staging (T1–T4), and different type of metastases (hepatic metastases for colon and gastric cancer and lymphnode metastases for melanoma). We observed an increase of FZD-10 protein in cytoplasm during the colon carcinogenesis in accordance to previously reported data about an up-regulation of RNA expression during colorectal carcinogenesis [12–13], suggesting a role as prognostic factor for this protein. For colorectal cancer, it is known that the Wnt signaling pathway is activated through the mutation of the adenomatous polyposis coli gene (APC) [10]. Usually the FDZ-10 proteins serve as receptors for Wnt that regulates the β-catenin pathway in oncogenesis of colon cancer. Wnt translocates into the cytosol and as part of a protein complex controls the degradation and translocation of β-catenin to the nucleus [11, 12, 14]. From our results, we assume that the FDZ-10 remains connected to the Wnt and becomes part of the protein complex translocating from the membrane to the nucleus in the early stages of tumor growth. The co-localization of Wnt and FDZ-10 will be studied in more detail in future.

For colon cancer, we observed a direct correlation between tumor staging and FZD-10 protein level in the cytoplasm while for skin and gastric cancer, the trend is opposite. Here we observed a decrease of FZD-10 in the cytoplasm with increasing malignancy of the tumor. In our opinion, this indicates that the Wnt pathway plays a minor role in gastric cancer and melanoma progression. However, the decrease of FDZ-10 signal in the nucleus with increasing malignancy was observed also in melanoma and to a lesser extent in gastric cancer.

For development of skin cancer it was reported that different Wnt isoforms are involved in epidermal carcinogenesis, in particular Wnt-7, as well as Wnt-2 and Wnt-5 [15]. The presence of FDZ-10 in our study confirms results by other authors who concluded that the FZD-10 is the receptor involved in the Wnt-7 cascade [15]. However, the role of Wnt in tumor progression is controversial [16–17] and needs a more detailed study because we demonstrated that FDZ-10 is absent in the nucleus at higher stages of the disease. The role of FDZ-10 is still unknown but the protein can be used as a marker for metastatic tumors, T3 and T4. The role of FDZ receptors in embryogenesis and its distribution in advanced tumors which usually are de-differentiated and advanced in endothelial mesenchymal transition could make the presence or absence of FDZ-10 an indicator for the differentiation level of the tumor cells.

## MATERIALS AND METHODS

### Patients

The present study is a retrospective analysis on paraffin-embedded tissue samples from Colon, Skin and Stomach, kindly provided by the Pathology laboratory, IRCCS Bari. All patients signed an informed consent. Details for each patient can be found in Supplementary Table 1 in Supplementary Material.

Colon tissues were obtained from 20 patients (M/F: 13/7, age 67 ± 9) who received a surgery for CRC and for three of them colonoscopy for diagnosis of polyps was performed. For 3 patients, we analyzed cancer tissue (1 patient with T1 cancer classification and 2 with T2 cancer classification), as well as associated polyps with low grade and high grade dysplasia.

Skin tissues were obtained from 29 patients (M/F: 14/15, age 58 ± 12), who underwent to excisional biopsy for dysplastic nevus and/or melanoma and metastases in the lymph nodes.

Gastric tissue was obtained from 20 patients (M/F: 10/10, age 69 ± 11) who received a surgery for GC or gastroscopy for non-pathological stomach issue. From 4 patients, we obtained additionally 2 samples of low dysplasia tissue and 2 samples of high dysplasia tissue, for this reasons we analyzed for 20 patients 24 samples.

Tissue samples were selected using morphological and histological criteria for each pathology (with TNM system), in order to obtain: A) adenomatous tissue, with low and high-grade dysplasia, adenocarcinoma and liver metastases for Colon, B) dysplastic, melanomatous tissue and lymph nodes metastases, for skin, C) adenomatous with low and high-grade dysplasia, carcinomatous and metastases for gastric tissue.

### Methods

In order to evaluate the pathology assessment, 4-mm thick sections were processed for histology and stained with hematoxylin-eosin staining (H&E), for each type of tissue (colon, skin and stomach). Successively, in normal, low- and high-grade dysplastic mucosa and in adeno-carcinomatous tissue for colon and stomach, and for normal, dysplastic and melanoma, FZD-10 expression was assessed by Immunohistochemistry.

### FZD-10 staining

In order to evaluate FZD-10 expression, an anti-Frizzled 10 polyclonal antibody recognizing the N-terminal part (AA sequence: HGKYEIPAQSPTCV; ab150564) was used. The investigation in the protein database SwissProt showed no similarities with any other protein for the recognized amino acid sequence. Briefly, after deparaffinization and antigen retrieval (shaking in Citrate buffer at pH 6.0 in a water bath at 98°C for 30 minutes) slides were treated with peroxidase blocking solution for 15 minutes and 3 times washed with PBS. Prior to the incubation with the primary antibody, the nonspecific sites were blocked by incubation with a BSA 1% in PBS solution for 2 hours, then the slides were incubated with primary antibodies (diluted 1:100 with phosphate buffered saline (PBS) and 5% FBS) overnight at 4°C. The anti-FZD-10 was detected with a polymer-based visualization kit (EnVision, DAKO A/S, Glostrup, Denmark), according to the manufacturer’s instructions, using 3,3-diaminobenzidine-tetrahydrochloride (DAB, Vector laboratories) as the chromogen, and nuclei were counterstained with Mayer’s hematoxylin (Sigma). FZD-10 immunoreactivity in cells was visible as a brown coloration of membranes, cytoplasm and/or nuclei. Positive and negative controls were included in each staining run, as indicated in the data sheet of antibody. For negative control, the primary antibody was omitted and replaced by PBS 1× pH 7.6.

### Immunohistochemical analysis

Specimens were examined under Olympus Bx 41 optical microscope (Olympus) at 20× and 40× magnification by a pathologist and the slides were acquired with a D-Sight slide scanner (Menarini Diagnostics srl). The images were processed for signal quantification with ImageJ. The staining assessment of FZD-10 was evaluated by counting the number of pixel (%) of immunoreactive tumor cells in more than 10 tumor areas at 40x magnification. For the evaluation of nuclear FZD-10, stained nuclei were count for more than 10 tumor areas for each analyzed slides at 40x magnification. Immunohistochemical assessment was always performed independently by two investigators.

### Statistical analysis

Statistical analyses were performed with Sigma-Stat 3.1 software by one-way analysis of variance (ANOVA). In critical cases, the Holm–Sidak method was applied for the comparison with the control group.

## SUPPLEMENTARY MATERIALS TABLES





## References

[1] Komiya Y, Habas R (2008). Wnt signal transduction pathways. Organogenesis.

[2] Logan CY, Nusse R (2004). The Wnt Signaling Pathway in Development and Disease. Annu Rev Cell Dev Biol.

[3] He X, Semenov M, Tamai K, Zeng X (2004). LDL receptor-related proteins 5 and 6 in Wnt/beta-catenin signaling: arrows point the way. Development.

[4] Schulte G, Bryja V (2007). The Frizzled family of unconventional G-protein-coupled receptors. Trends Pharmacol Sci.

[5] Wallingford JB, Habas R (2005). The developmental biology of Dishevelled: an enigmatic protein governing cell fate and cell polarity. Development.

[6] Koike J, Takagi A, Miwa T, Hirai M, Terada M, Katoh M (1999). Molecular cloning of Frizzled-10, a novel member of the Frizzled gene family. Biochem Biophys Res Commun.

[7] Nagayama S, Fukukawa C, Katagiri T, Okamoto T, Aoyama T, Oyaizu N, Imamura M, Toguchida J, Nakamura Y (2005). Therapeutic potential of antibodies against FZD10, a cell-surface protein, for synovial sarcomas. Oncogene.

[8] Fukukawa C, Hanaoka H, Nagayama S, Tsunoda T, Toguchida J, Endo K, Nakamura Y, Katagiri T (2008). Radioimmunotherapy of human synovial sarcoma using a monoclonal antibody against FZD10. Cancer Sci.

[9] Itzkowitz SH, Harpaz N (2004). Diagnosis and management of dysplasia in patients with inflammatory bowel diseases. Gastroenterology.

[10] Nagayama S, Yamada E, Kohno Y, Aoyama T, Fukukawa C, Kubo H, Watanabe G, Katagiri T, Nakamura Y, Sakai Y, Toguchida J (2009). Inverse correlation of the up-regulation of FZD10 expression and the activation of b-catenin in synchronous colorectal tumors. Cancer Sci.

[11] Huang HC, Klein PS (2004). The Frizzled family: receptors for multiple signal transduction pathways. Genome Biology.

[12] Terasaki H, Saitoh T, Shiokawa K, Katoh M (2002). Frizzled-10, up-regulated in primary colorectal cancer, is a positive regulator of the WNT - beta-catenin - TCF signaling pathway. Int J Mol Med.

[13] Najdi R, Holcombe RF, Waterman ML (2011). Wnt signaling and colon carcinogenesis: Beyond APC. J Carcinog.

[14] Peifer M, Polakis P (2000). Wnt signaling in oncogenesis and embryogenesis: a look outside the nucleus. Science.

[15] Pham K, Milovanovic T, Barr RJ, Truong T, Holcombe RF (2003). Wnt ligand expression in malignant melanoma: pilot study indicating correlation with histopathological features. J Clin Pathol: Mol Pathol.

[16] Lim X, Nusse R (2013). Wnt signaling in skin development, homeostasis, and disease. Cold Spring Harb Perspect Bio.

[17] Webster MR, Weeraratna AT (2013). A Wnt-er migration: the confusing role of β-cateninin melanoma metastasis. Sci Signal.

