# Supplementary material for: Frizzled-10 and cancer progression: Is it a new prognostic marker?
Source: Oncotarget. 2017 Dec 12;9(1):824–30. doi: 10.18632/oncotarget.23159 (PMC5787514; doi:10.18632/oncotarget.23159)
Supplement: Supplementary file 1 [file oncotarget-09-824-s001.pdf]

Frizzled-10 and cancer progression: Is it a new prognostic marker?

SUPPLEMENTARY MATERIALS

Supplementary Table 1: Data of the single patients. See Supplementary_Table_1

Supplementary Table 2: Data analysis for the different analysed pathologies

Type of tissue	Colon		Skin		Stomach	
Pathological diagnosis	% Positive pixel cytoplasm	% Positive nuclei	% Positive pixel cytoplasm	% Positive nuclei	% Positive pixel cytoplasm	% Positive nuclei
DYSPLASTIC NEVUS			67.89 ± 25.23	94.73 ± 12.56		
Hyperplasia	10.45 ± 5.2	78.89 ± 23.1				
LOW GRADE DYSPLASIA	29.43 ± 13.15	74.57 ± 10.4			33.76 ± 0.25	70.47 ± 7.07
HIGH GRADE DYSPLASIA	36.41 ± 20.16	59.32 ± 15.60			20.44 ± 1.42	56.94 ± 25.33
Tis TUMOR			89.2 ± 22.12	71.76 ± 10.98		
T1 TUMOR	36.78 ± 10.51	54.23 ± 5.6	77.26 ± 5.12	95.91 ± 23.15	20.99 ± 9.19	44.66 ± 25.95
T2 TUMOR	55.06 ± 20.47	40.67 ± 18.65	55.29 ± 4.18	86.07 ± 9.54	21.14 ± 7.54	63.24 ± 7.3
T3 TUMOR	65.18 ± 16.29	5.04 ± 2.96	78.63 ± 9.29	3.44 ± 0.26	18.5 ± 0.21	40.11 ± 19.22
T4 TUMOR	44.25 ± 11.21	3.38 ± 1.24	32.64 ± 2.23	2.56 ± 1.1	11.5 ± 1.77	28.6 ± 13.09
METASTASES	68.24 ± 13.23	1.69 ± 0.053	38.42 ± 3.58	6.89 ± 1.45	7.99 ± 3.33	9.82 ± 7.25
